# Consequences of the multipatient use of a single-patient capillary blood sampling device (CBSD)

**DOI:** 10.1186/1753-6561-5-S6-P92

**Published:** 2011-06-29

**Authors:** P Staeger, F Ninane, L Mazzolai, D Moradpour, J Wasserfallen, E Masserey, L Senn, G Zanetti

**Affiliations:** 1 University Hospital, Lausanne, Switzerland; 2 Service of Public Health, Lausanne, Switzerland

## Introduction / objectives

Multipatient use of a single-patient CBSD occurred in an outpatient clinic during 4 to 16 months before its notification. We looked for transmission of blood-borne pathogens among exposed patients.

## Methods

Exposed patients underwent serology testing for HBV, HCV and HIV. Patients with isolated anti-HBc received one dose of hepatitis B vaccine to look for a memory immune response. Possible transmissions were investigated by mapping visits and sequencing of the viral genome if needed.

## Results

Of 280 exposed patients, 9 had died without suspicion of blood-borne infection, 3 could not be tested, and 5 declined investigations. Among the 263 (93%) tested patients, 218 (83%) had negative results. We confirmed a known history of HCV infection in 6 patients (1 co-infected by HIV), and also identified resolved HBV infection in 37 patients, of whom 18 were already known. 2 patients were found to have a previously unknown HCV infection. According to the time elapsed from the closest previous visit of a HCV-infected potential source patient, we could rule out nosocomial transmission in one case (14 weeks) but not in the other (1 day). In the latter, however, transmission was deemed very unlikely by 2 reference centers based on the sequences of the E1 and HVR1 regions of the virus.

## Conclusion

We did not identify any transmission of blood-borne pathogens in 263 patients exposed to a single-patient CBSD, despite the presence of potential source cases. Change of needle and disinfection of the device between patients may have contributed to this outcome. Although we cannot exclude transmission of HBV, previous acquisition in endemic countries is a more likely explanation in this multi-national population.

## Disclosure of interest

None declared.

